# Depression in the Spousally Bereaved Elderly: Correlations with Subjective Sleep Measures

**DOI:** 10.1155/2013/409538

**Published:** 2013-03-24

**Authors:** Timothy H. Monk, Marissa K. Pfoff, Joette R. Zarotney

**Affiliations:** ^1^Department of Psychiatry, University of Pittsburgh, 3811 O'Hara Street, Pittsburgh, PA 15213, USA; ^2^University of Pittsburgh School of Medicine, 3550 Terrace Street, Pittsburgh, PA 15261, USA

## Abstract

Complaints of poor sleep and symptoms of depression are likely to coexist in the spousally bereaved elderly. This study was concerned with the correlation between depressive symptoms and various measures of subjectively reported sleep using questionnaire and diary instruments in 38 bereaved seniors (60y+). Correlations between the sleep measures and *days since loss* and grief intensity were also calculated. All sleep disruption measures correlated significantly with depression score, but only sleep duration correlated with grief intensity, and no sleep measure correlated with *days since loss*. Therapies which address both sleep and depression are likely to be of benefit to bereaved seniors.

## 1. Introduction

The elderly are especially vulnerable to depression, partly due to endogenous factors linked to the aging process [1]. Superimposed upon this, though, is the fact that at some time in the older person's life the individual's spouse may die, leaving them spousally bereaved. Spousal bereavement is a very devastating, life-altering event that becomes more likely with advancing age [2, 3]. Widowhood affects millions of men, and a greater number of women, around the world every year. 

Immediately after the loss of a close loved one, people experience a period of acute grief that generally includes intrusive thoughts, intense emotional distress, and withdrawal from normal daily activities [4]. This period, along with the chronic grief experience that follows, may vary in length and intensity from individual to individual and often resembles clinical depression [5]. Major depression afflicts about 28% of the spousally bereaved [6]. This risk of depression appears to peak during the first six months of bereavement [2, 7], although depressive symptoms can be present for up to 2 years [8]. Even bereaved persons with subclinical levels of depressive symptomatology may suffer extensively, for they too have a greater likelihood of functional impairment, poorer health, more physician visits and mental health counseling, and increased use of antidepressants than do nonbereaved individuals [3, 9, 10].

In a parallel manner, elderly persons are (again, probably for endogenous reasons) particularly vulnerable to sleep disruption [11, 12]. Also, bereavement *per se* has been shown to interfere with sleep [10, 13]. Thus, depressive symptoms and complaints of poor sleep are likely to coexist in the spousally bereaved elderly. This study was concerned with the correlation between depressive symptoms (as measured by the Hamilton Rating Scale for Depression (HRSD) [14]), and various measures of subjectively reported sleep using questionnaire and diary instruments [15, 16]. Correlations were also tested between the sleep measures and *days since loss* as well as the grief score from the Texas Revised Index of Grief (TRIG). Thirty-eight spousally bereaved seniors were studied starting no sooner than two months after bereavement, before commencing a 6-month treatment study. Other aspects of the study have been and will be reported elsewhere [17, 18]. The study aimed to determine whether the sleep disruption associated with bereavement was primarily associated with H1: the level of depressive symptomatology; H2: the level of grief severity; and/or H3: the time since loss. The nature of these interrelationships is important and is relevant to the current vigorous debate concerning grief in DSM-V [19]. 

## 2. Materials and Methods

### 2.1. Subjects

Recruitment was by word of mouth, advertising, and presentations and meetings with appropriate groups. A total of 38 subjects (aged 60y+, 30 f, 8 m) were studied, starting no sooner than two months after spousal bereavement (range 76 to 498 days, mean 220 days). Subjects were not currently taking antidepressants and were in a stable medical condition (see below). 

### 2.2. Medical Screening

Medical Screening on all subjects took place prior to the first therapy session and included a complete medical history and physical examination, including a review of current medical records from subjects' personal physicians. We included only subjects with stable, nonacute chronic medical problems, such as well-controlled hypertension or hypothyroidism. Potential subjects who exhibited acute symptoms warranting treatment were not included until a stable treatment regimen had been implemented. Medical screening also included a routine laboratory panel (complete blood count, electrolytes, blood glucose, BUN, creatinine, liver function tests, thyroid function tests, urinalysis, and electrocardiogram). 

### 2.3. Psychiatric Screening

Within 2 weeks prior to the start of therapy, a psychiatric screening was conducted to exclude potential subjects who were suffering from psychotic disorder, or substance abuse disorder as determined by administration of the Structured Clinical Interview for DSM-IV. Subjects were also required to score 24 or greater on the Folstein Mini-mental State Examination to exclude patients with dementia. Subjects currently taking antidepressants were excluded.

### 2.4. Sleep Screening

Within 2 weeks prior to the start of therapy, each subject attended for one recorded night of sleep (full montage polysomnography with oximetry) in order to rule out subjects with an Apnea Hypopnea Index (AHI) >30. 

### 2.5. Procedure

The present study was concerned with measures collected from subjects prior to starting a major study involving 6-month course of therapy and the collection of extensive measures at various time-points (reported elsewhere [17]). Depressive symptoms were measured using Hamilton Rating Scale for Depression (HRSD) [14], as administered by a master's or doctoral level therapist. The HRSD was scored after excluding the questions pertaining to sleep. The Pittsburgh Sleep Quality Index (PSQI) and the Texas Revised Index of Grief (TRIG) instruments were also given. The PSQI is a widely used questionnaire of subjective sleep quality yielding a score between 0 and 21, with higher numbers indicating more problems, and a score of >5 suggesting poor sleep [20]. The TRIG [21] is widely used to measure grief, yielding a numerical score with higher numbers denoting more grief. There was also given a field evaluation involving completion of a Pittsburgh Sleep Diary (PghSD) every night for 2 consecutive weeks [16]. From this, the following subjective sleep measures (14 night averages) were derived: (1) time spent asleep (TSA) and (2) percent sleep efficiency (SE—the percentage of the time in bed spent actually asleep). Demographic information was obtained, and subjects carefully and tactfully questioned to ascertain the exact date of their spouse's death, so the *days since loss* measure could be calculated. A battery of pencil-and-paper tests and questionnaires related to mental health, physical health, activity, functioning, performance, and well-being was also given, as were laboratory-based measures of sleep and circadian rhythms at various time-points throughout the 6-month study (reported in Monk et al. [17, 18] and elsewhere). 

### 2.6. Statistical Analysis 

The predictor variables were HRSD score, TRIG score, and *days since loss*. The dependent variables were PSQI score and the two measures derived from the 2-week sleep diary (TSA, SE). Correlations coefficients were calculated using the nonparametric Spearman rho statistic. A significance level of *P* < 0.05 (2-tailed) was applied. 

## 3. Results and Discussion

Mean values (with s.d.) of the predictor and dependent variables are given in Table 1. Data loss was small and related to subjects misunderstanding or not answering questions: 0 subjects for HRSD, 2 for TRIG, 4 for PSQI, and 1 for the PghSD measures. The correlations are reported in Table 2. 

Table 1 reveals that this sample showed some sleep disruption and a below average sleep efficiency, netting only 6.6 hours of sleep per night, which is close to the 25th percentile value found for 1166 seniors (65y+) in our recent telephone survey study [22]. Because higher scores on the PSQI represent *worse* sleep, the pattern of correlations (Table 2) was consistent in showing a worsening of sleep with increasing depressive symptoms. For TRIG score, there was a significant correlation with TSA (shorter sleep associated with greater grief), but no correlation with the other sleep measures (PSQI, SE). No significant correlations emerged between *days since loss* and any of the sleep measures. Within the predictor variables, TRIG score only very weakly correlated with HRSD score (rho = 0.286, *P* = 0.09). There was no correlation between *days since loss* and HRSD score (rho = 0.021, n.s.) and only a very weak correlation between *days since loss* and TRIG score (rho = 0.262, *P* = 0.13).

The pattern of results confirmed Hypothesis H1 of the study in indicating that those widow(er)s with the most depressive symptoms also showed the worse sleep, as measured by both questionnaire and diary. The fact that sleep for these two instruments is essentially “at home” is important. One possible etiology for widow(er)s' sleep disruption is simply the loss of a loved one who may have been the subject's cohabitant for many decades and whose absence at night might be particularly painful. Also, many widows feel a lessened sense of security at night in the absence of their husbands. Neither of these issues would be as potent in a sleep laboratory setting.

Hypothesis H2 (regarding the correlation between sleep and grief level) was confirmed for TSA, but not for PSQI or SE. This dissociation between the three sleep measures in their relation to grief severity is important and worthy of further study. It is noteworthy that, in the literature, SE and PSQI appear to be more closely related to the patient's own subjective feelings about their sleep than does sleep duration *per se *[23, 24]. The lack of any correlation between sleep and *days since loss* indicates that Hypothesis H3 was not confirmed. 

The strength and generality of HRSD as a predictor of sleep bespeaks the importance of depressive symptomatology in late-life bereavement. This is in line with earlier findings from Reynolds and colleagues which have indicated the importance of both clinical [13] and subclinical [10] depression in the sleep of the bereaved elderly. With regards to clinical depression, Reynolds et al. [13] showed that the sleep of depressed bereaved subjects was almost identical to that of nonbereaved depressed unipolar depressed patients of matched age and gender. In analyzing the possible cognitive mechanism for this sleep disruption in the spousally bereaved elderly, Hall et al. [25] showed that levels of intrusive thoughts and avoidance behaviors reported by depressed bereaved subjects were extremely high, resembling values reported among individuals with posttraumatic stress disorder. 

Although the link between depression and insomnia in bereaved seniors is compelling, it must be recognized that the design of the study precludes testing the *direction* of the association, and it may equally be the case that the poor sleep exacerbates the depressive symptoms (remembering that the questions pertaining to sleep in the HRSD were excluded in this analysis). Should that be the case, then improvements in sleep either by pharmacological or behavioral therapy might alleviate the depressive symptoms so often felt by the elderly bereaved. Whatever the direction of causality, therapies which address both sleep and depression are likely to be of benefit to bereaved seniors. 

## 4. Conclusions

In bereaved seniors, symptoms of depression and disruptions in sleep are likely to coexist. In the present sample, both questionnaire and diary-based measures of sleep correlated significantly with level of depressive symptomatology. Sleep duration correlated with the level of grief severity, but sleep efficiency and subjective sleep quality (PSQI) did not. No sleep measure correlated with *days since loss*.

## Figures and Tables

**Table 1:** Sample mean and standard deviation (s.d.) for each of the three predictor variables and three dependent variables. The sample size of 38 is reduced slightly for some variables due to missing data (see text).

Variable	Mean	s.d.
HRSD (depression)	5.47	4.47
TRIG (grief)	53.22	12.71
Days since loss	220	86
PSQI (sleep quality index)	6.32	3.51
TSA (time spent asleep)	6.6 h	0.9 h
SE (percent sleep efficiency)	84.3%	6.6%

**Table 2:** Spearman rho correlation coefficients. Significance is denoted: *P < 0.05, **P < 0.01, ***P < 0.001 (2-tailed). Sample size of 38 was reduced slightly for some comparisons because of missing data (see text).

Variable	PSQI	Time spent asleep	Sleep efficiency
HRSD	0.550***	−0.422**	−0.655***
Days since loss	−0.272	−0.257	−0.028
TRIG score	0.092	−0.610***	−0.276
